# 3-D solar cells by electrochemical-deposited Se layer as extremely-thin absorber and hole conducting layer on nanocrystalline TiO_2_ electrode

**DOI:** 10.1186/1556-276X-8-8

**Published:** 2013-01-03

**Authors:** Duy-Cuong Nguyen, Souichirou Tanaka, Hitoshi Nishino, Kyohei Manabe, Seigo Ito

**Affiliations:** 1 Department of Electric Engineering and Computer Sciences, University of Hyogo, Shosha 2167, Himeji, Hyogo, 671-2280, Japan; 2 Energy Technology Laboratories, Osaka Gas Co. Ltd, 6-19-9, Torishima, Konohana-Ku, Osaka, 554-0051, Japan

**Keywords:** 3-D solar cells, Nanocrystalline TiO_2_ electrode, Se layer

## Abstract

A three-dimensional selenium solar cell with the structure of Au/Se/porous TiO_2_/compact TiO_2_/fluorine-doped tin oxide-coated glass plates was fabricated by an electrochemical deposition method of selenium, which can work for the extremely thin light absorber and the hole-conducting layer. The effect of experimental conditions, such as HCl and H_2_SeO_3_ in an electrochemical solution and TiO_2_ particle size of porous layers, was optimized. This kind of solar cell did not use any buffer layer between an n-type electrode (porous TiO_2_) and a p-type absorber layer (selenium). The crystallinity of the selenium after annealing at 200°C for 3 min in the air was significantly improved. The cells with a selenium layer deposited at concentrations of HCl = 11.5 mM and H_2_SeO_3_ = 20 mM showed the best performance, resulting in 1- to 2-nm thickness of the Se layer, short-circuit photocurrent density of 8.7 mA/cm^2^, open-circuit voltage of 0.65 V, fill factor of 0.53, and conversion efficiency of 3.0%.

## Background

Three-dimensional (3-D) solar cells were developed by Nanu et al. and O'Hayre et al. [1-4]. The structure of these solar cells is similar to dye-sensitized solar cells (DSCs) [5-8]; however, this kind of 3-D solar cell does not use a liquid electrolyte like DSC. Hence, 3-D solar cells can get better stability than DSCs. The other advantage of 3-D solar cells is a short migration distance of the minority carriers and, therefore, reduces the recombination of electrons and holes [3]. In addition, 3-D solar cells are easily fabricated by non-vacuum methods such as spray pyrolysis and chemical bath depositions; consequently, they are well-known as low cost solar cells. The major photoabsorber materials in the 3-D compound solar cells have been CuInS_2_[1-4,9], CuInSe_2_[10], Se [11], Sb_2_S_3_[12-17], CdSe [18,19], and CdTe [20,21]. In the 3-D compound solar cells, the buffer layer between the TiO_2_ and absorber layer was commonly utilized to block charge recombination between electrons in TiO_2_ and holes in hole-transport materials [1-4,9,10,12-16].

In this paper, we study 3-D solar cells using selenium for the light absorber layer. Selenium is a p-type semiconductor with a band gap of 1.8 and 2 eV for crystal and amorphous states, respectively. Flat selenium solar cells were researched by Nakada in the mid-1980s [22,23]. The selenium solar cells with a superstrate structure showed the best efficiency of 5.01% under AM 1.5 G illumination. In our work, the selenium layer was prepared by electrochemical deposition (ECD), a non-vacuum method, resulting in the extremely thin absorber (ETA) [11-21]. The similarly structured solar cells (3-D selenium ETA solar cells deposited on nanocrystalline TiO_2_ electrodes using electrochemical deposition) were also studied by Tennakone et al. [11], which were composed with hole-conducting layer of CuSCN. The Se layer worked just to be a photoabsorber.

In this report, on the other hand, the 3-D Se ETA solar cells worked without a CuSCN layer. We did not use any buffer layers between the n-type electrode porous TiO_2_ and the selenium photoabsorber layer, or any additional hole-conducting layer. Hence, the Se layer worked bi-functionally as photoabsorber and hole conductor. The effect of the TiO_2_ particle size, HCl and H_2_SeO_3_ concentrations, and annealing temperature on the microstructure and photovoltaic performance was investigated thoroughly.

## Methods

**Figure 1:**
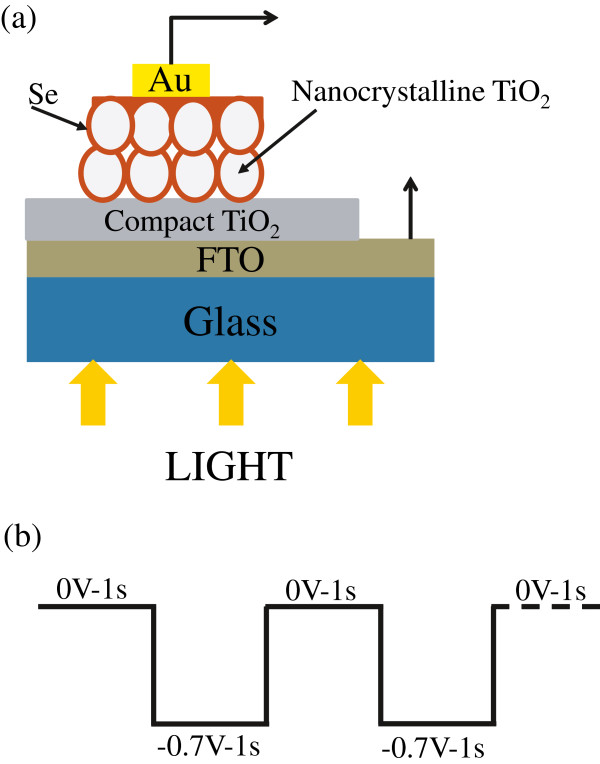
**The 3**-**D solar cell structure and the electrochemical deposition.** <Au/Se/porous TiO_2_/compact TiO_2_/fluorine-doped tin oxide-coated glass plates > **(a)** and the voltage pulse pattern for the electrochemical deposition of Se **(b)**.

The structure of the 3-D selenium ETA solar cell was described in Figure 1a. Transparent conducting oxides of fluorine-doped tin oxide (FTO)-coated glass plates (TEC-7, Nippon Sheet Glass Co., Ltd., Tokyo, Japan; *t* = 2.2 mm) were used as substrates. The 70-nm TiO_2_ compact layer was prepared at 400°C in air by a spray pyrolysis deposition method. The solution used for depositing the TiO_2_ compact layer was a mixture of titanium acetylacetonate (TAA) and an ethanol with ethanol/TAA volume ratio of 9:1. The TAA solution was prepared by the slow injection of acetylacetone (purity of 99.5%, Kanto Chemical Co., Inc., Tokyo, Japan) into titanium tetraisopropoxide (purity of 97%, Kanto Chemical Co., Inc.) with a mole ratio of 2:1. After TiO_2_ compact layer deposition, samples were immersed into a 40 mM aqueous TiCl_4_ aqueous solution at 70°C for 30 min for the purpose of removing pin holes in TiO_2_ compact layers and washed with water and ethanol. The porous TiO_2_ layers with different TiO_2_ particle sizes were coated by a screen-printing method. The TiO_2_ particles were ST21 (Ishihara Sangyo Kaisha, Ltd., Osaka, Japan) for *d* = 20 nm, F-2 (Showa Titanium Co., Ltd., Toyama, Japan) for *d* = 60 nm, F-1 (Showa Titanium Co., Ltd.) for *d* = 90 nm, and ST41 (Ishihara Sangyo Kaisha, Ltd., Japan) for *d* = 200 nm. The thickness of porous TiO_2_ layers was fixed at 2 μm. The detail about preparing the TiO_2_ paste and sintering after screen printing was described in the previous report [24]. Selenium absorber layers were deposited for 20 min by the ECD method. The solution for ECD includes 0.45 M NaCl (purity of 99.5%, Kanto Chemical Co., Inc.), HCl (concentration of 20 w/w%, Kishida Chemical Co., Ltd., Osaka, Japan), and H_2_SeO_3_ (purity of 97%, Kanto Chemical Co., Inc.); the water was used as solvent. The concentrations of HCl and H_2_SeO_3_ were discussed in the ‘Results and discussions’ section. The pulse potential (on-off) was applied during ECD. The pulse potential was described in Figure 1b. Ag/AgCl (BAS Inc., Tokyo, Japan) was used as a reference electrode. The total voltage-applying duration and the total off time are 10 min each. Hence, the total deposition duration (including off time) was 20 min. All samples after depositing by ECD were annealed at 200°C for 3 min in the air to improve the crystallinity of selenium layers. After the annealing, the 3-D selenium ETA solar cells were completed with gold electrodes deposited by an evaporation method. The area of cells for the photocurrent density-voltage (J-V) measurement is 0.25 cm^2^.

In order to confirm the crystallinity of selenium before and after annealing, X-ray diffraction (XRD) (Mini Flex II, Rigaku Corporation, Tokyo, Japan) was carried out. The cross-section and surface morphology of the samples were measured by scanning electron microscopy (SEM) (JSM-6510, JEOL Ltd., Tokyo, Japan). The coverage on nanocrystalline TiO_2_ by Se was observed by high resolutiontransmission electron microscopy (JEM 2100 F, JEOL Ltd.). Absorption spectra were measured by an ultraviolet–visible spectroscopy (Lambda 750 UV/VIS spectrometer, PerkinElmer Inc., MA, USA). Photovoltaic measurements employed an AM 1.5 G solar simulator equipped with a xenon lamp (YSS-80, Yamashita Denso Corporation, Tokyo, Japan). The power of the simulated light was calibrated to 100 mW cm^−2^ using a reference Si photodiode (Bunkoukeiki Co., Ltd., Tokyo, Japan). J-V curves were obtained by applying an external bias to the cell and measuring  the  generated  photocurrent  with  a  DC  voltage  current source (6240A, ADCMT Corporation, Tokyo, Japan).

## Results and discussion

**Figure 2:**
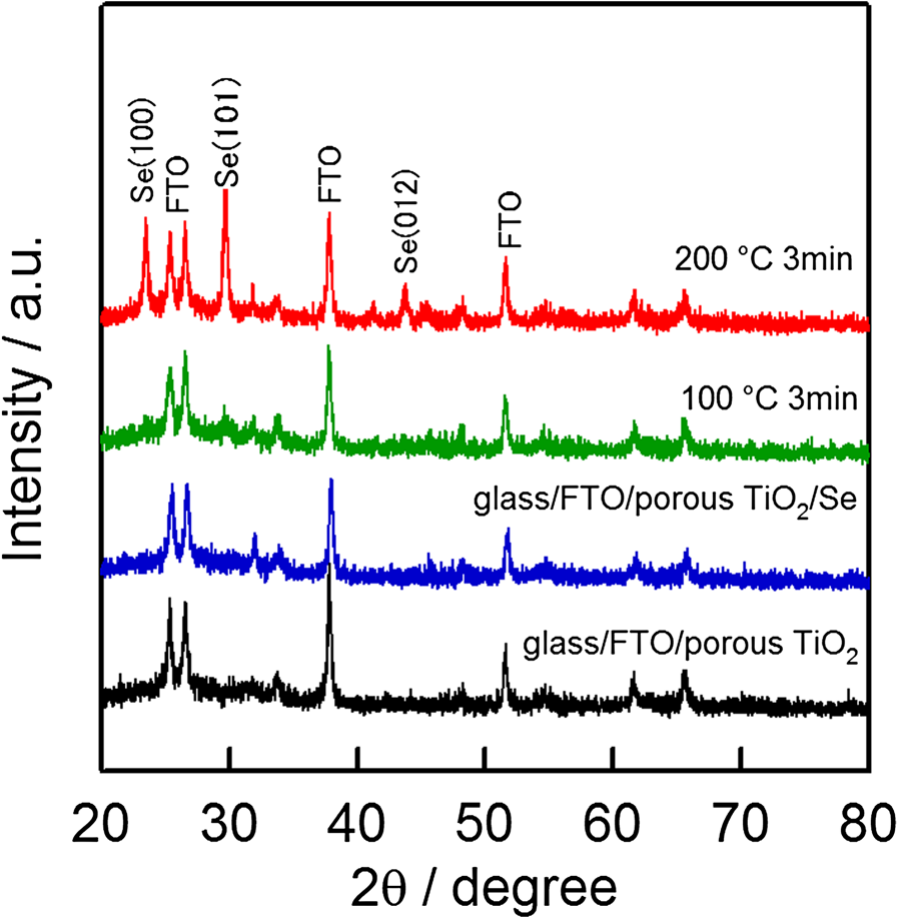
**The XRD patterns of porous TiO**_**2**_/**compact TiO**_**2**_/**FTO with**/**without Se electrochemical deposition and with**/**without annealing.**

In order to improve the crystallinity of the selenium layer, the samples after ECD were annealed at different temperatures. Figure 2 shows the XRD pattern of selenium depositing on porous TiO_2_/compact TiO_2_/FTO/glass before and after annealing at various temperatures for 3 min in the air. The XRD peaks of selenium were not observed at an as-deposition sample. This indicates that the selenium layer was in an amorphous state. In the case of the sample annealing at 100°C, a weak peak of selenium was observed at the position of 29.6°; this means that the improvement of the crystallinity in selenium was insignificant. However, when the annealing temperature of Se was increased to 200°C, strong peaks were observed at the positions of 23.5°, 29.7°, and 43.8°, and these peaks were indexed at (100), (101), and (012) of selenium, respectively [25]. The appearance of Se strong peaks at the sample annealing at 200°C indicates a strong improvement of the crystallinity in the selenium absorber layer. The change in the crystallinity of selenium will cause an effect on the optical and microstructural properties, as well as on photovoltaic performance. This topic will be discussed in more detail in the absorption spectra, SEM image, and photocurrent density-voltage results below.

**Figure 3:**
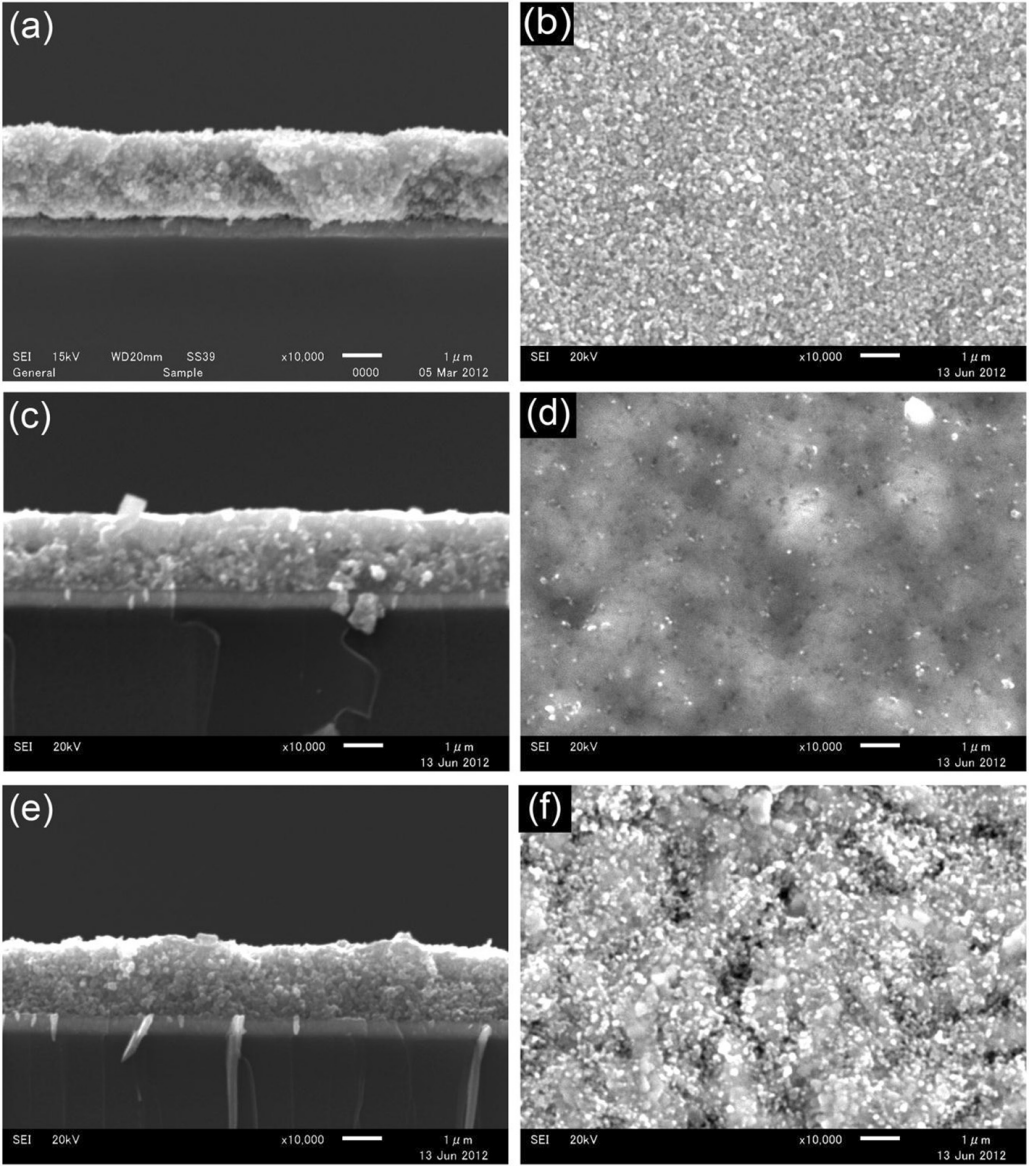
**SEM images of cross**-**sections and surface annealings.** Cross-section **(a)** and surface **(b)** of the porous TiO_2_/compact TiO_2_/FTO/glass, the cross-section **(c)** and surface **(d)** of Se-coated porous TiO_2_ before annealing, and the cross-section **(e)** and surface** (f) **of Se-coated porous TiO_2_ after annealing at 200°C for 3 min.

Figure 3 shows the cross-sectional and surface SEM images of porous TiO_2_, Se-coated porous TiO_2_ without annealing, and Se-coated porous TiO_2_ with annealing at 200°C for 3 min in the air. From the cross-sectional images, as shown in Figure 3a,c,e, it is difficult to recognize the changes in the microstructure in the samples before and after depositing selenium, as well as with and without annealing.

The surface of porous TiO_2_ is rather rough (see Figure 3b) because the particle size of TiO_2_ nanoparticles is big, approximately 60 nm. However, the surface became smoother after depositing selenium as shown in Figure 3d. Figure 3f shows the surface morphology of selenium-coated porous TiO_2_ after annealing at 200°C for 3 min in the air. The surface is rougher than that of before annealing. Big particles were observed in this sample. The appearance of big particles and a rough surface is due to the improvement of the crystallinity of selenium after annealing, as mentioned in the XRD section above.

**Figure 4:**
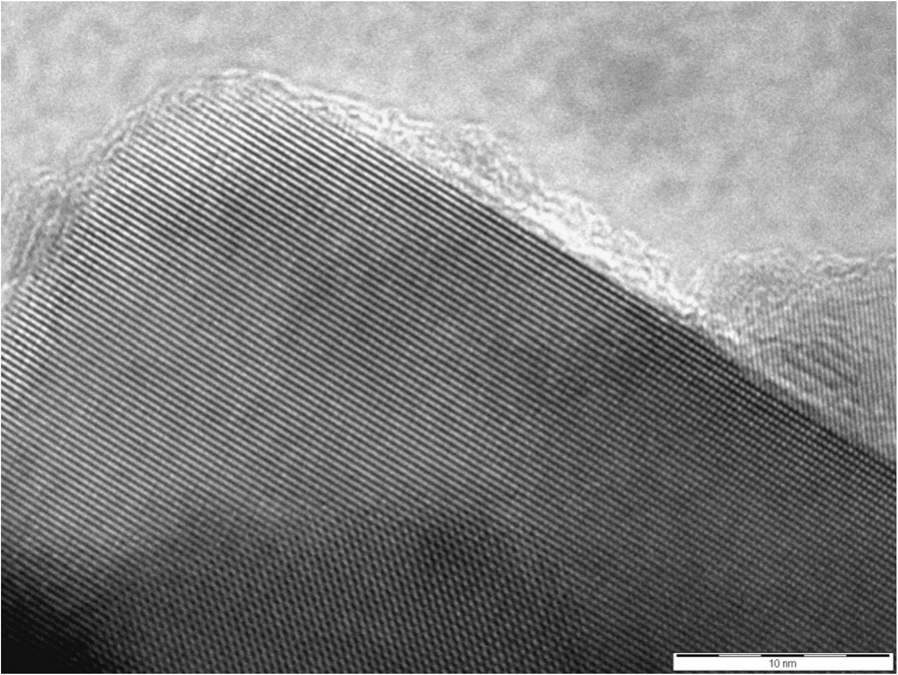
**A TEM image of the Se**-**deposited nanocrystal TiO**_**2**_**electrode after annealing at 200**°**C.**

Figure 4 shows a TEM image of Se-deposited TiO_2_ nanocrystals after annealing at 200°C, which was observed after receiving a scratching from an FTO glass substrate and deposited on a Cu grid for TEM. It was confirmed that an extremely thin electrodeposited Se layer (*t* = 1 to 2 nm) existed on TiO_2_ nanoparticles. Since the Se layer is very thin, it should function in two ways: the photoabsorber and the hole conductor, as illustrated in Figure 1a.

**Figure 5:**
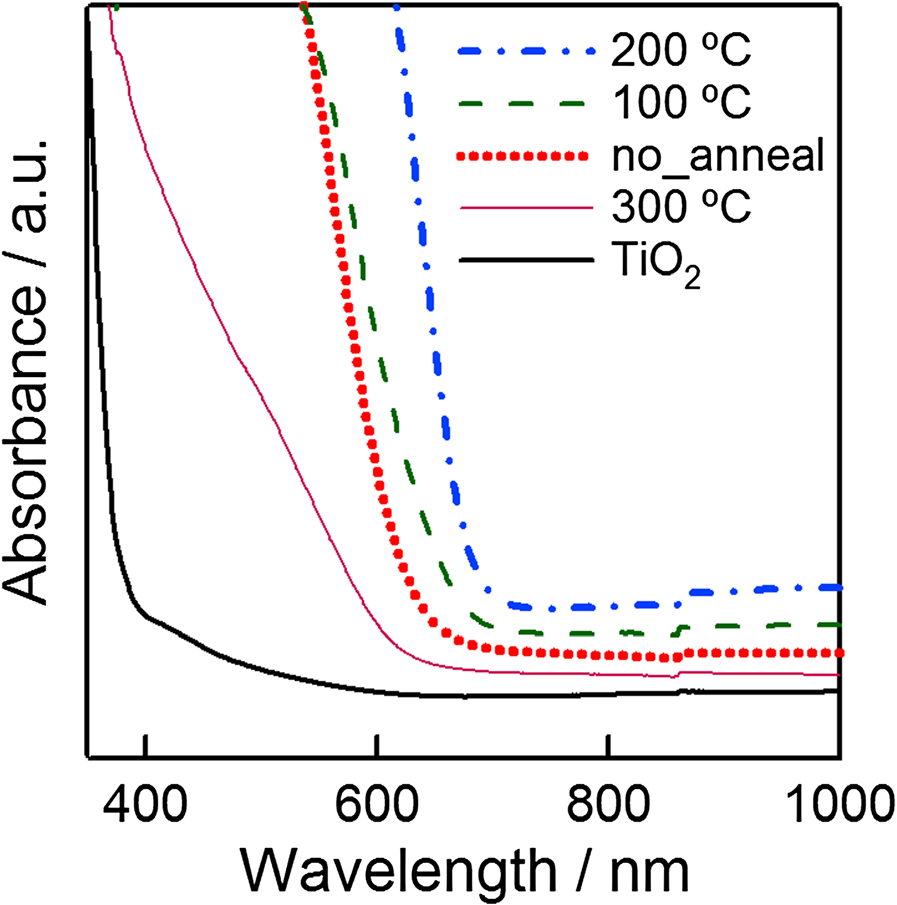
**The absorption spectra of selenium with**/**without annealing at various temperatures under air.**

Figure 5 depicts the absorption spectra of Se-coated porous TiO_2_ without annealing and with annealing at 100°C, 200°C, and 300°C. The band gap of as-deposited Se is 2.0 eV; this is the band gap of amorphous selenium. After annealing, the absorption edges were shifted towards a longer wavelength. The band gaps of the sample annealed at 100°C and 200°C are 1.9 and 1.8 eV, respectively. The fact that the band gap of selenium becomes narrower after annealing may be attributed to the increase in crystallinity as mentioned in the XRD and SEM results. When the annealing temperature was increased up to 300°C, the absorption edge shifted towards a shorter wavelength. The light absorption of 300°C-annealed Se became lower in comparison to selenium with and without annealing at 100°C and 200°C. The decrease in the light absorption of selenium may be due to the fact that a part of selenium escaped from the sample during annealing because the melting point of selenium is quite low, approximate 217°C [23]. From the absorption spectra and XRD results, the sample annealed at 200°C for 3 min in the air was inferred to be the best condition.

**Figure 6:**
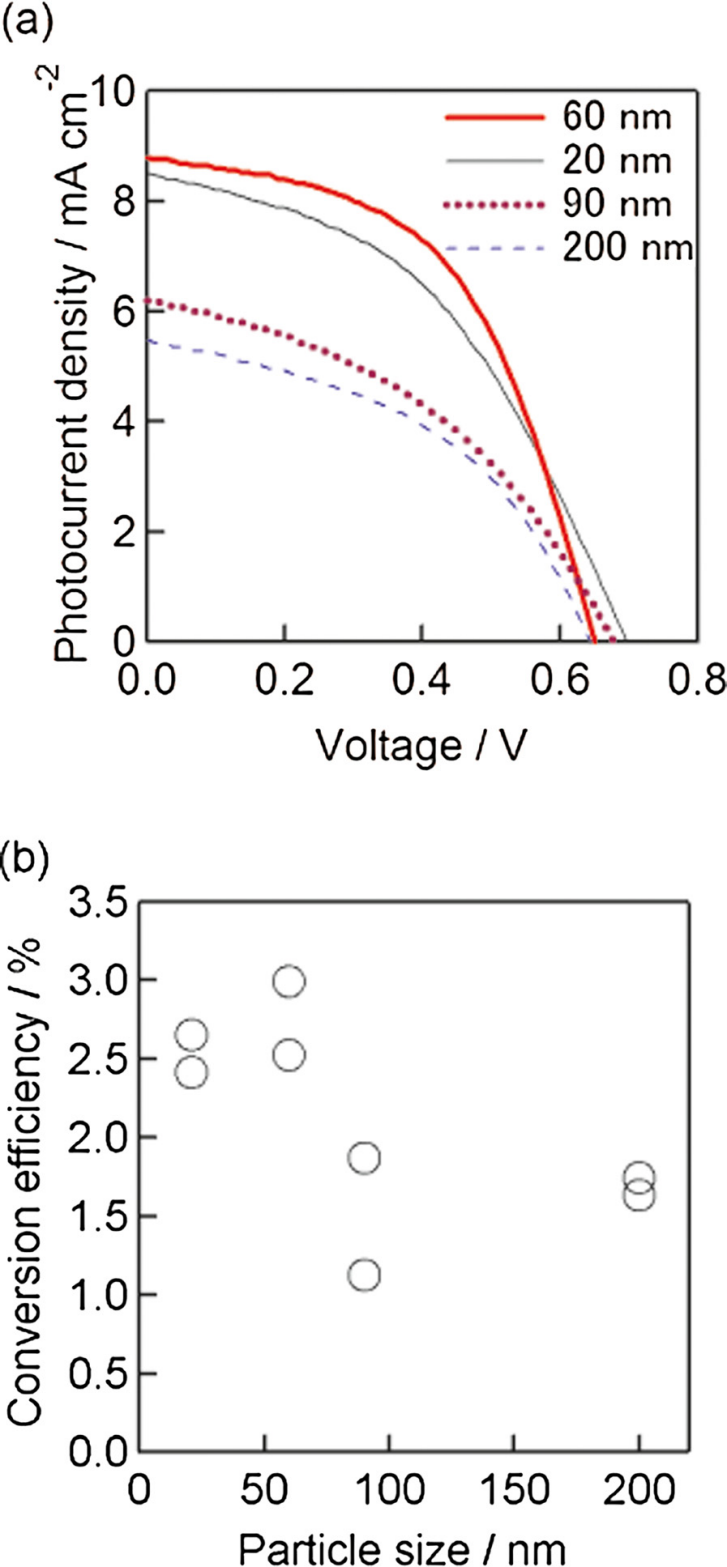
**Photocurrent density**-**voltage curves and variation of conversion efficiency.** Photocurrent density-voltage curves of 3-D selenium ETA solar cells **(a)** and the variation of conversion efficiency **(b)** with different TiO_2_ particle sizes used for the porous TiO_2_ layer. The annotation numbers in Figure 6a suggest the sizes of the nanocrystalline TiO_2_ particle utilized for the electrodes.

In order to optimize the particle size of TiO_2_ nanoparticles for the porous layer, 3-D selenium ETA cells were fabricated with different TiO_2_ nanoparticle sizes. Figure 6 shows the photocurrent density-voltage curves and the variation of the conversion efficiency of 3-D selenium ETA cells with various TiO_2_ particle sizes. The concentrations of HCl and H_2_SeO_3_ were kept at 11 and 20 mM, respectively. The cells fabricated with 90 and 200 nm TiO_2_ particles showed lower photocurrents (*J*_SC_ = 5.5 and 6.2 mA/cm^2^ for 200 and 90 nm TiO_2_, respectively). The best cell was observed in the sample using 60-nm TiO_2_ nanoparticles for the porous layer. Hence, 60-nm TiO_2_ nanoparticles are optimal for fabricating the porous layer. The parameters of the best cells are short-circuit photocurrent density (*J*_SC_) = 8.7 mA/cm^2^, open-voltage (*V*_OC_) = 0.65 V, fill factor (FF) = 0.53, and conversion efficiency (*η*) = 3.0%. The variation of conversion efficiency is shown in Figure 6b. The efficiency decreased with the increase in the TiO_2_ particle size over 60 nm. The low performance of solar cells with 20-nm TiO_2_ nanocrystallites can be explained by small pores, and therefore, it was difficult to deposit Se inside the porous TiO_2_ layer. In the case of the TiO_2_ particle size being over 60 nm, the decrease of cell performance may be due to the bad connection between nanocrystalline TiO_2_ particles; the connection between nanocrystalline TiO_2_ particles is better with a smaller particle size at the same sintering temperature.

**Figure 7:**
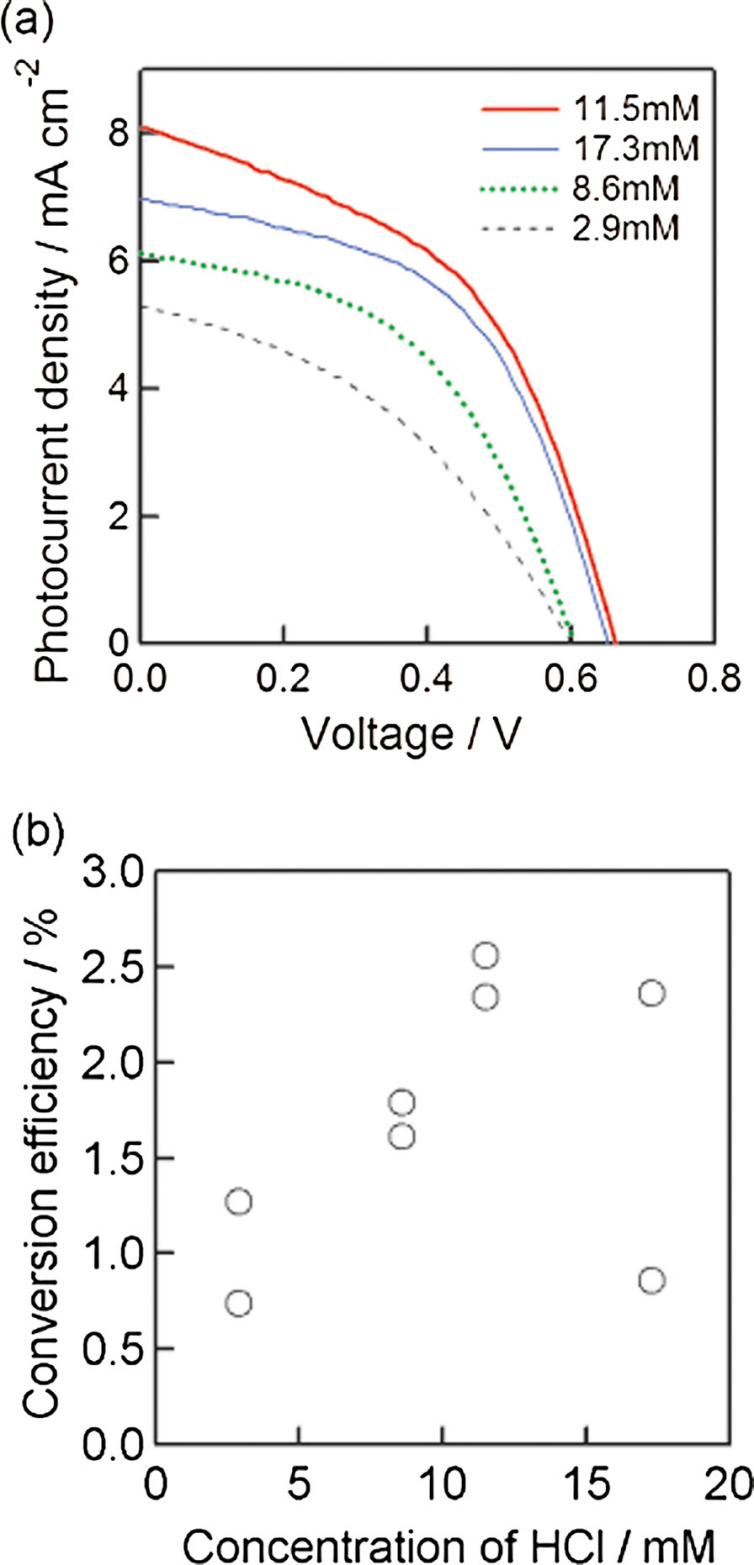
**Photocurrent density**-**voltage curves and variation of the conversion efficiency of 3**-**D selenium ETA solar cells.** Photocurrent density-voltage curves **(a)** and the variation of conversion efficiency **(b)** of 3-D selenium ETA solar cells with different HCl concentrations. The annotation numbers in Figure 7a suggest the HCl concentrations for Se deposition.

Figure 7 shows the photocurrent density-voltage curves and the variation of the conversion efficiency of 3-D selenium ETA solar cells with HCl concentrations in the solution for depositing selenium. The TiO_2_ nanoparticle with a 60-nm diameter was utilized for the porous layer, and the concentration of H_2_SeO_3_ was kept at 20 mM. From Figure 6a, the photocurrent density increased with the increase in HCl concentration in the range of 2.9 to 11.5 mM and decreased with HCl concentration of over 11.5 mM. The cells deposited at HCl concentrations of 11.5 and 17.3 mM showed a higher *V*_OC_ than those that were prepared at 2.9 and 8.6 mM HCl. Figure 6b shows the variation of the conversion efficiency with an HCl concentration in the ECD solution. The highest conversion efficiency was obtained at the concentration of 11.5 mM. In the case of samples deposited with the concentrations of 2.9 and 8.6 mM HCl, Se was almost observed at the outer porous TiO_2_; this is the reason for getting a low cell performance. Conversely, Se distributed uniformly from the bottom to the top of porous TiO_2_ at an HCl concentration of 11.5 mM. Further addition of HCl (17.3 mM) caused the deposition rate of Se to become rather fast and the porous-TiO_2_ layer to easily break and fall off from the substrate; this can explain the low cell performance of samples depositing at 17.3 mM HCl.

**Figure 8:**
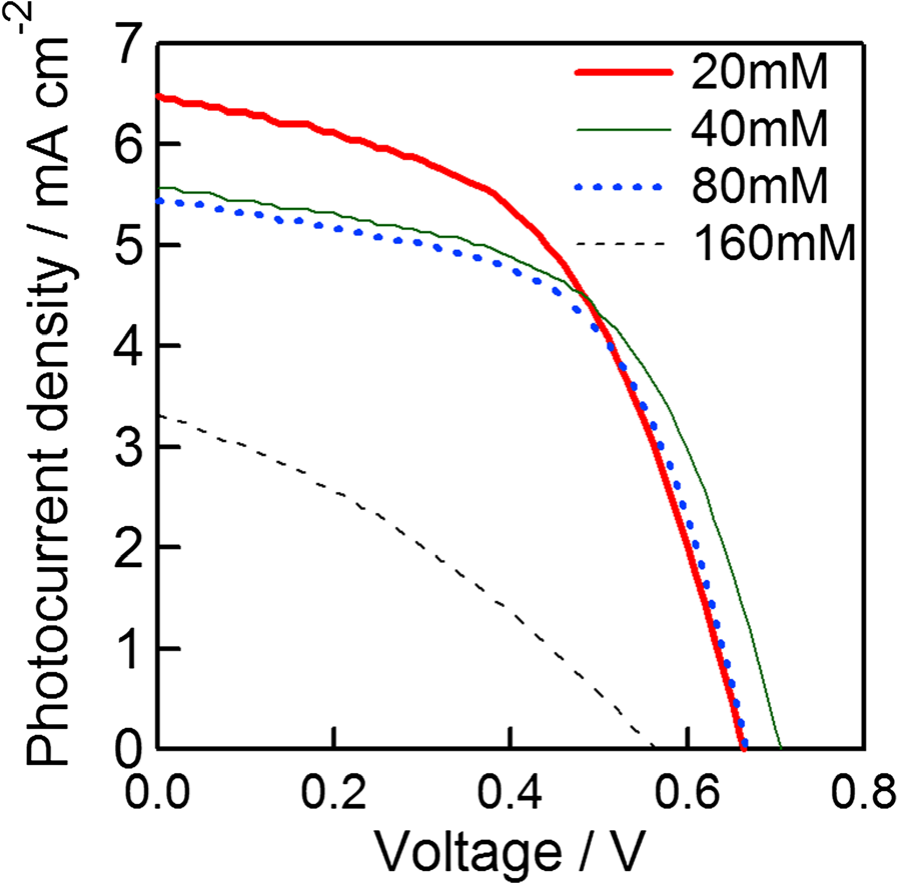
**Photocurrent density**-**voltage curves of selenium solar cells with various H**_**2**_**SeO**_**3**_**concentrations. **The annotation numbers in Figure 8 suggest the H_2_SeO_3_ concentrations.

In order to investigate the effect of H_2_SeO_3_ concentration on the cell performance, cells were prepared at various H_2_SeO_3_ concentrations. Figure 8 depicts the photocurrent density-voltage curves with different H_2_SeO_3_ concentrations. The HCl concentration in these experiments was kept at 11.5 mM, and 60-nm TiO_2_ nanoparticles were utilized for the porous layer. From the results, the photovoltaic performance of cells is seemingly better at a lower H_2_SeO_3_ concentration. The best cell performance was observed at 20 mM H_2_SeO_3_. When the concentration of H_2_SeO_3_ was over 20 mM, the deposition rate was rather fast, so the porous TiO_2_ layers easily broke and peeled off. This may be the reason behind the low cell performance.

## Conclusion

3-D selenium ETA solar cells using an extremely thin absorber Se layer on nanocrystalline TiO_2_ electrodes were fabricated by electrochemical deposition method. The crystallinity of the selenium layer after annealing at 200°C for 3 min in the air was significantly improved, and the band gap became narrower in comparison to the sample both with and without annealing at 100°C. The photovoltaic performance features of the best 3-D selenium ETA solar cells are *J*_SC_ = 8.7 mA/cm^2^, *V*_OC_ = 0.65 V, FF = 0.53, and *η* = 3.0%. These results are interesting for PV researchers because the fabrication method for this kind of solar cells is quite simple. However, in order to get a higher efficiency, the photocurrent density should be more improved.

## Competing interest

The authors declare that they have no competing interests.

## Authors’ contributions

DCN organized and wrote the manuscript. ST proposed the original data and carried out the fabrication of TiO_2_ electrode, the deposition of Se by electroplating, and the measurement of photovoltaic results. HN, KM and SI were the supervisors of the research. All authors read and approved the final manuscript.
